# Smoking and injury severity score predict major complications after surgical treatment of ipsilateral proximal and shaft femoral fractures

**DOI:** 10.1051/sicotj/2026072

**Published:** 2026-10-15

**Authors:** Luis Henrique Longo, Weverley Rubele Valenza, Paulo Henrique Vogt, Lucas Romano Sampaio Rezende, Matheus Uriel Senna Klipp, Henrique Rezende Corral, Lucas Cruz Cepeda, Christiano Barbieri de Oliveira Martoni

**Affiliations:** Department of Orthopedics and Traumatology, Complexo Hospitalar do Trabalhador Curitiba Paraná Brazil

**Keywords:** Femoral fractures, Multiple trauma, Smoking, Injury severity score, Postoperative complications

## Abstract

*Introduction*: Ipsilateral proximal and shaft femoral fractures are rare, high-energy injuries associated with substantial morbidity. Evidence on predictors of adverse outcomes after surgical treatment remains limited. *Methods*: We conducted a retrospective cohort study at a tertiary trauma center including all skeletally mature patients who underwent surgical treatment for ipsilateral proximal femoral fractures (femoral neck or trochanteric region) associated with femoral shaft fractures between January 2019 and December 2024. The primary outcome was a composite of major complications, including deep surgical site infection, delayed union, nonunion, symptomatic pulmonary embolism, or all-cause mortality. Bivariable analyses were performed to compare patients with and without major complications, followed by multivariable logistic regression to identify independent predictors. *Results*: Fifty-two patients were included, with a mean age of 41.8 years and a mean Injury Severity Score (ISS) of 23.1. Major complications occurred in 17 patients (32.7%). The most frequent major complications were all-cause mortality (13.5%) and deep surgical site infection (11.5%). In bivariable analysis, smoking, longer time to definitive treatment, and higher ISS were associated with major complications. In the multivariable model, smoking (adjusted odds ratio [OR] 19.24, 95% confidence interval [CI] 2.78–133.04; *p* = 0.003) and higher ISS (adjusted OR per point 1.10, 95% CI 1.01–1.19; *p* = 0.027) remained independently associated with the primary outcome, whereas time to definitive treatment and age did not. *Conclusion*: Major complications were frequent after surgical treatment of ipsilateral proximal and shaft femoral fractures, affecting nearly one-third of patients. Smoking and greater trauma severity, as measured by ISS, were the main independent predictors of adverse outcomes. These findings highlight the importance of perioperative risk stratification and support smoking as a relevant modifiable risk factor in this setting.

## Introduction

Ipsilateral proximal and femoral shaft fractures occur predominantly in young patients sustaining high-energy trauma and are frequently associated with other fractures and visceral injuries [1]. It is estimated that proximal femoral fractures coexist in up to 10% of patients with ipsilateral femoral shaft fractures [2], with approximately 75% of cases occurring in males, most commonly around the age of 35 years [3].

First described in 1953 by Delaney and Street, this combined injury pattern has shown increasing incidence, particularly in association with motorcycle-related trauma [4, 5]. The femoral shaft fracture is typically comminuted and located in the mid-diaphysis, and may be open in 15%–33% of cases [1]. In contrast, the proximal fracture is often basocervical, vertically oriented, and minimally displaced, which may lead to it being overlooked during initial assessment; up to 30% of these proximal fractures may not be identified at hospital admission, highlighting the importance of maintaining a high index of diagnostic suspicion [1, 3].

The severity of polytrauma in these patients is significant. Up to 75% may present with associated visceral or systemic injuries, and 20%–40% may have concomitant injuries involving the lower extremity [6].

The management of these combined fractures remains challenging, with no clear consensus regarding the optimal fixation strategy [7, 8]. Although locked intramedullary nailing is the standard treatment for femoral shaft fractures and proximal fractures may be treated with cannulated screws, dynamic hip screws (DHS), or cephalomedullary nails, the debate between single versus dual implants remains unresolved [8]. Early surgical treatment is generally recommended to reduce morbidity and mortality and to facilitate mobilization [3]; however, in polytrauma settings, delays may be unavoidable depending on prioritization of life-threatening injuries.

Understanding the occurrence of complications – such as surgical site infection, delayed union, nonunion, reoperation, and thromboembolism – and their associated factors is essential, as these events directly reflect the impact of high-energy trauma and the complexity of patient care [9].

Therefore, the primary objective of this study was to identify factors associated with the occurrence of complications in patients with surgically treated ipsilateral proximal and femoral shaft fractures.

## Materials and methods

This study was conducted and reported in accordance with the Strengthening the Reporting of Observational Studies in Epidemiology (STROBE) guidelines for cohort studies [10], as shown in the flow diagram in Figure 1.

**Figure 1:**
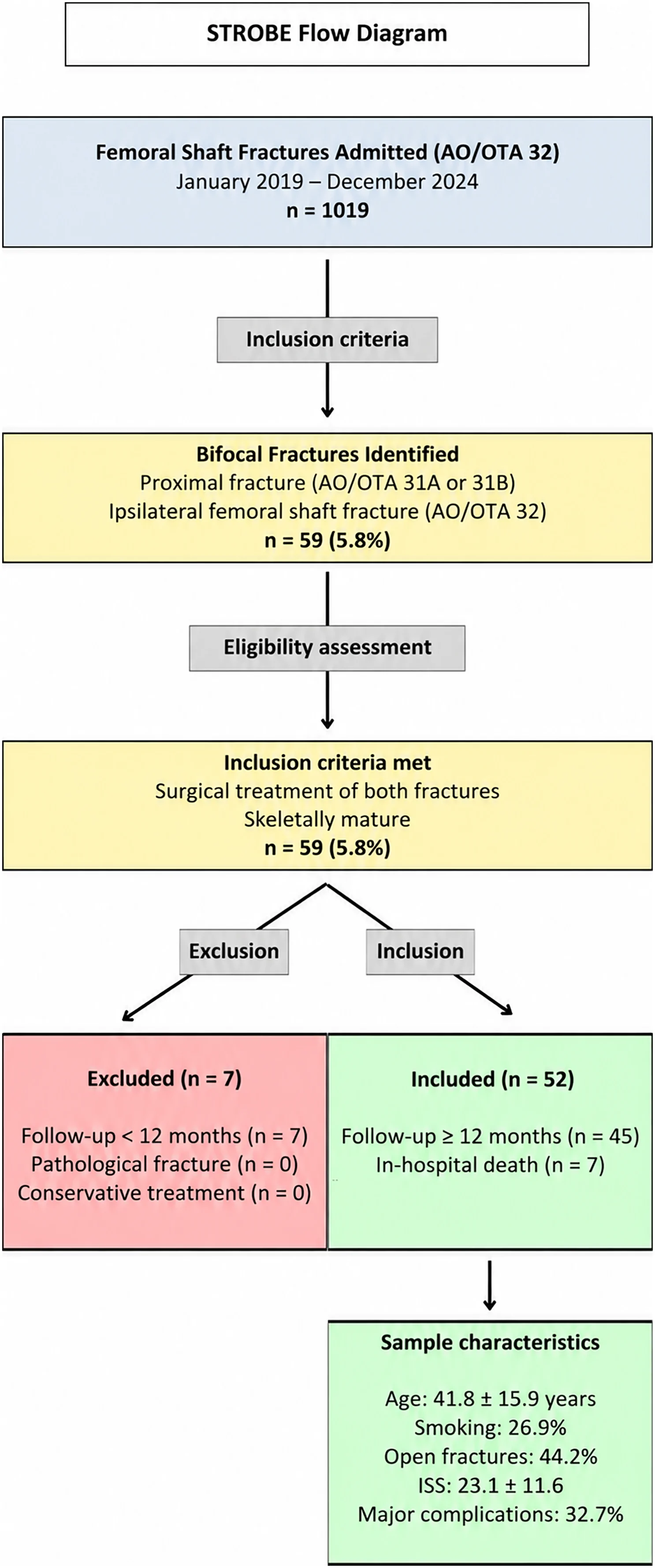
STROBE flow diagram of patient selection.

### Study population and data collection

Skeletally mature patients with a confirmed diagnosis of ipsilateral bifocal femoral fracture (a femoral shaft fracture associated with a proximal femoral fracture, AO/OTA 31A or 31B [11]) were included if they underwent surgical treatment for both injuries and had a minimum follow-up of 12 months. Cases of pathological fractures, non-surgical treatment, or follow-up shorter than 12 months (except for in-hospital deaths) were excluded. Data were retrospectively collected from electronic medical records and the institutional PACS system.

Definitive fixation during the initial surgical procedure was performed when clinical and local conditions allowed. When staged management was selected on a case-by-case basis, temporary external fixation was used as a damage-control orthopedic measure. No formal standardized damage-control orthopedics protocol was in place, and the decision was individualized according to the overall injury burden, competing surgical priorities, soft-tissue conditions, and clinical status.

All patients with open fractures received antibiotic therapy and operative irrigation and debridement during initial management.

### Outcomes and variables

The primary outcome was a composite of major complications: deep surgical site infection (deep SSI) requiring surgical debridement; delayed union, defined as absence of callus formation in three cortices after 6 months; nonunion, defined as absence of fracture healing after 9 months requiring revision surgery; symptomatic and confirmed pulmonary embolism (PE); or all-cause mortality. Secondary outcomes included superficial surgical site infection (superficial SSI), deep vein thrombosis (DVT), and unplanned reoperation.

Independent variables collected included demographic characteristics (age, sex), lifestyle factors (smoking), comorbidities, and trauma-related details (mechanism of injury, Injury Severity Score – ISS [12], open fracture according to the Gustilo–Anderson classification [13], associated injuries), as well as fracture characteristics (AO/OTA classification) and treatment variables (time to surgery and type of implant).

Outcomes of the proximal femoral fracture were assessed separately according to fracture pattern (femoral neck, AO/OTA 31B; or intertrochanteric, AO/OTA 31A). Clinical records and available radiographs were reviewed for proximal fracture union, delayed union, nonunion, femoral head avascular necrosis, varus malunion, loss of reduction, implant failure or cut-out, and reoperation attributable to a proximal fracture complication. Patients who died were excluded from the denominator used to assess late radiographic outcomes.

### Statistical analysis

Statistical analysis was performed using R software (version 4.3.1), with a significance level set at *p* < 0.05. After descriptive analysis, chi-square or Fisher’s exact tests were used for categorical variables, and Student’s *t*-test or Mann–Whitney *U* test for continuous variables in the bivariable analysis, comparing patients with and without the primary outcome.

Variables with *p* < 0.20 in the bivariable analysis were simultaneously entered into a multivariable logistic regression model to identify independent predictors. Results were expressed as odds ratios (ORs) with 95% confidence intervals (CIs). Model performance was assessed using the Hosmer–Lemeshow test and Nagelkerke’s pseudo R^2^. The association between open fracture status and deep SSI was specifically assessed using a 2 × 2 contingency table and a two-sided Fisher’s exact test because of the small number of events. The conditional OR and exact 95% CI were calculated. Analyses of the remaining secondary outcomes followed the same bivariable approach.

### Ethical considerations

The study was approved by the local Research Ethics Committee (CAAE: 86099725.8.0000.5225), with waiver of informed consent. Data confidentiality was ensured.

## Results

From January 2019 to December 2024, 1019 patients with femoral shaft fractures were identified at a level-1 trauma center. Of these, 59 patients (5.8%) presented with an associated ipsilateral proximal femur fracture. Seven patients were excluded due to loss to follow-up, resulting in a final sample of 52 patients.

The population was predominantly young (mean age 41.8 ± 15.9 years; median 42 years) and male (92.3%). The sample reflected high-severity trauma, with a mean ISS of 23.1 ± 11.6. Motorcycle accidents predominated (82.7%), followed by motor vehicle accidents (7.7%), falls (5.8%), pedestrian injury (1.9%), and gunshot wounds (1.9%). Open fractures occurred in 44.2%, mostly Gustilo-Anderson type II (23.1%). Associated fractures were present in 36.5%, notably leg bone fractures (17.3%), tibial plateau fractures (7.7%), and patellar fractures (5.8%).

The mean time from admission to definitive treatment was 8.7 ± 5.8 days (median, 8.5 days; range, 1–21 days). Nine patients (17.3%) underwent definitive fixation as part of the initial surgical management. The remaining 43 patients (82.7%) were managed with a staged strategy, using temporary external fixation followed by definitive fixation after a mean of 10.3 ± 5.0 days (median, 11 days; range, 2–21 days). All 23 patients with open fractures received antibiotic therapy and operative irrigation and debridement. Decisions regarding staged treatment were individualized according to the overall injury burden, competing surgical priorities, soft-tissue conditions, and operating-room prioritization at a high-volume tertiary trauma referral center.

Comorbidities were documented in 21.2%, with hypertension being most frequent (13.5%). Smoking was reported in 26.9%. The ISS distribution is shown in Figure 2.

**Figure 2:**
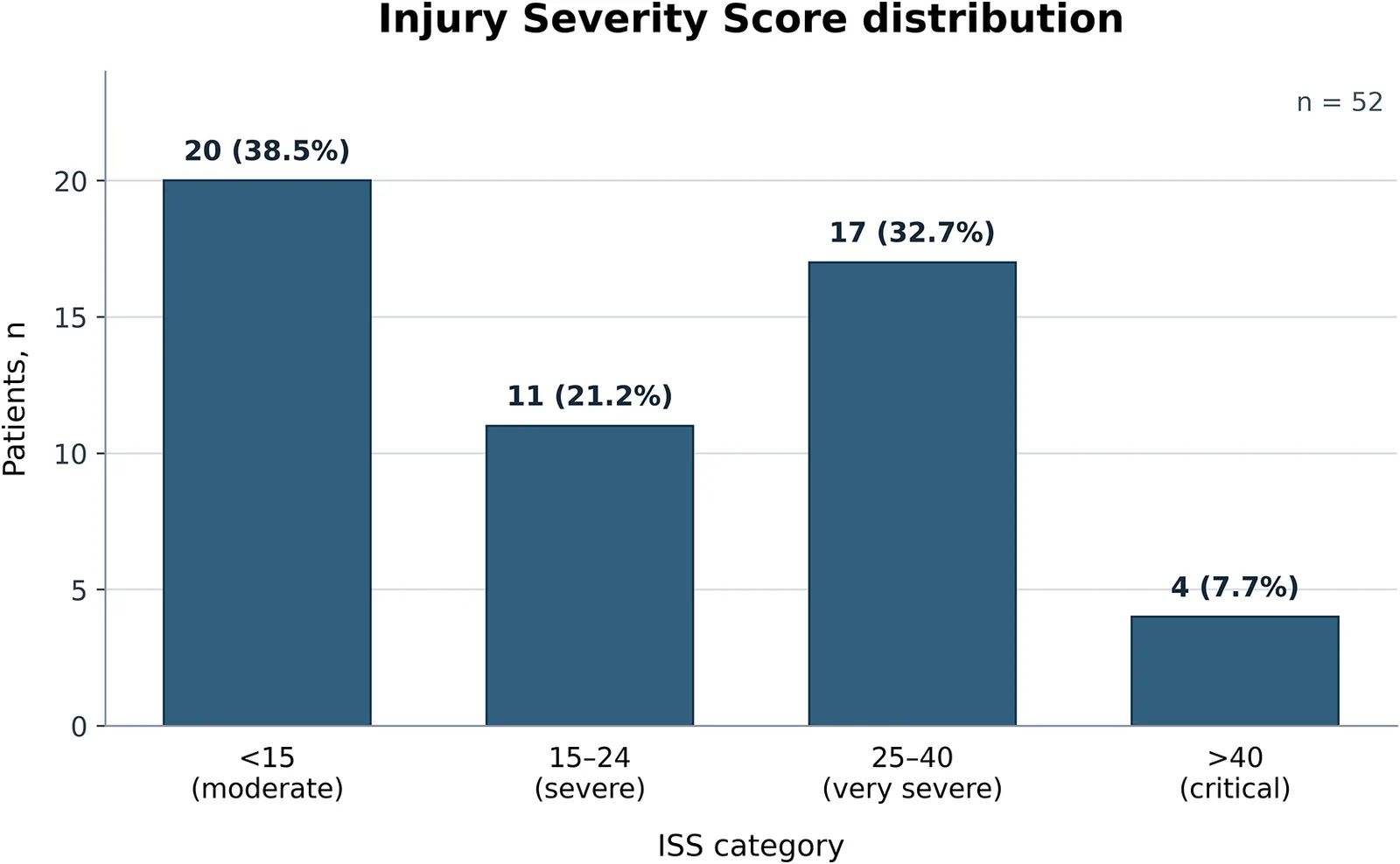
Distribution of Injury Severity Score (ISS) categories in the study population. Bars represent the number and percentage of patients in each category (*n* = 52).

Proximal femur neck-type fractures (AO/OTA 31B) represented 53.8% of cases, with 60.7% classified as basicervical (31B3) and 39.3% as transcervical (31B2). Intertrochanteric fractures (AO/OTA 31A) comprised 46.2%, predominantly simple fractures (31A1) in 66.7%, followed by multifragmented (31A2) and reverse oblique (31A3) types at 16.7% each. The proximal fracture classification distribution is shown in Figure 3.

**Figure 3:**
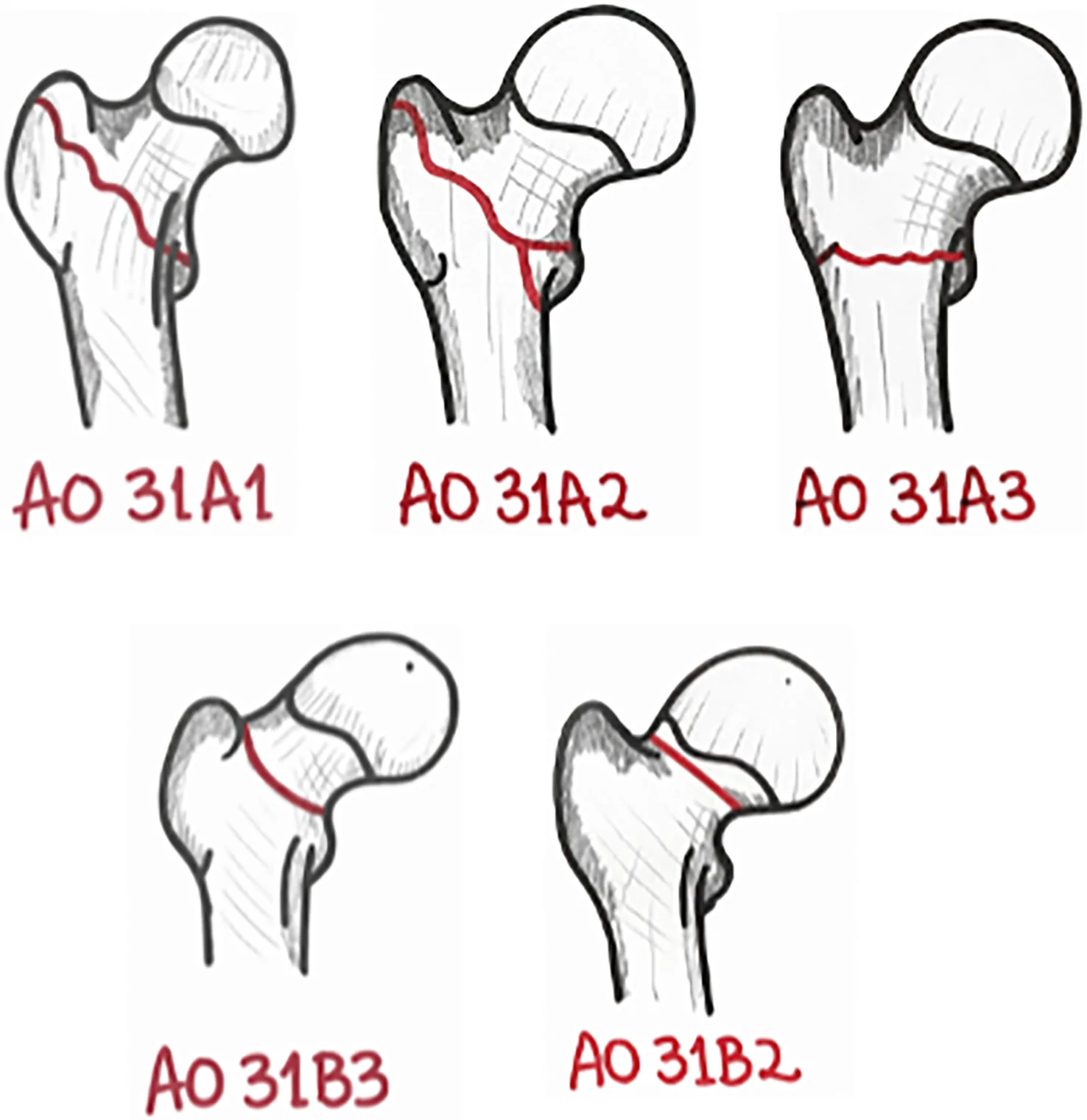
Distribution of proximal femoral fractures according to the AO/OTA classification. Intertrochanteric fractures (*n* = 24): AO 31A1 (*n* = 16), AO 31A2 (*n* = 4), and AO 31A3 (*n* = 4). Femoral neck fractures (*n* = 28): AO 31B3 (*n* = 17) and AO 31B2 (*n* = 11).

Femoral shaft fractures predominantly exhibited the AO 32A3 pattern (75.0%). Wedge fractures (AO 32B) represented 21.2%, with intact wedges (32B2) comprising 81.8% and multifragmented wedges (32B3) at 18.2%. Segmental fractures (AO 32C) were rare (3.8%). The shaft fracture classification is reproduced in Figure 4.

**Figure 4:**
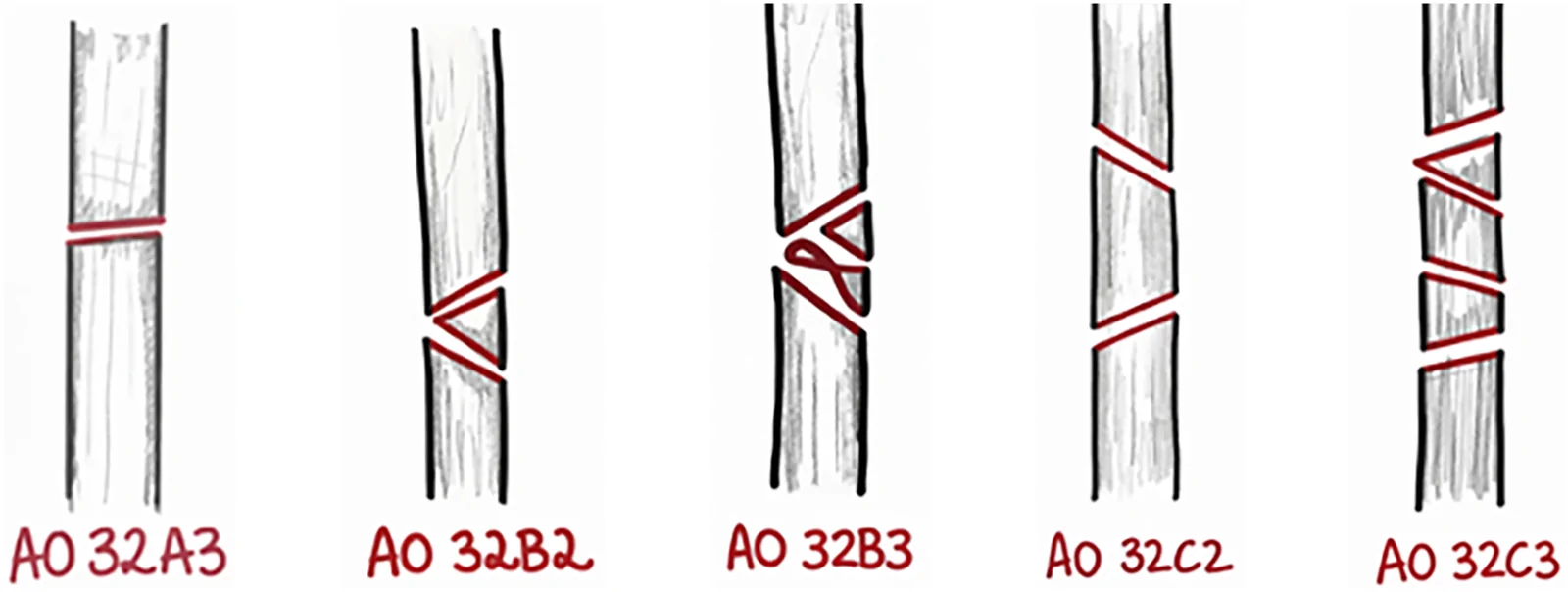
Distribution of femoral shaft fractures according to the AO/OTA classification. Femoral shaft fractures: AO 32A3 (*n* = 39), AO 32B2 (*n* = 9), AO 32B3 (*n* = 2), AO 32C2 (*n* = 1), and AO 32C3 (*n* = 1).

For proximal fracture treatment, the DHSs was most commonly used (51.9%), with anti-rotational screw augmentation in 13.5% of cases to enhance rotational stability. Long cephalomedullary nailing was employed in 25.0% for simultaneous stabilization of both fractures through a single implant, and cannulated screws in 9.6%. For shaft fractures, retrograde femoral nailing (DFN) predominated (75.0%), with long cephalomedullary nailing in 25.0%. Augmentation with a 3.5 mm DCP plate was used in only 3.8% of cases. Treatment strategies are shown in Figure 5.

**Figure 5:**
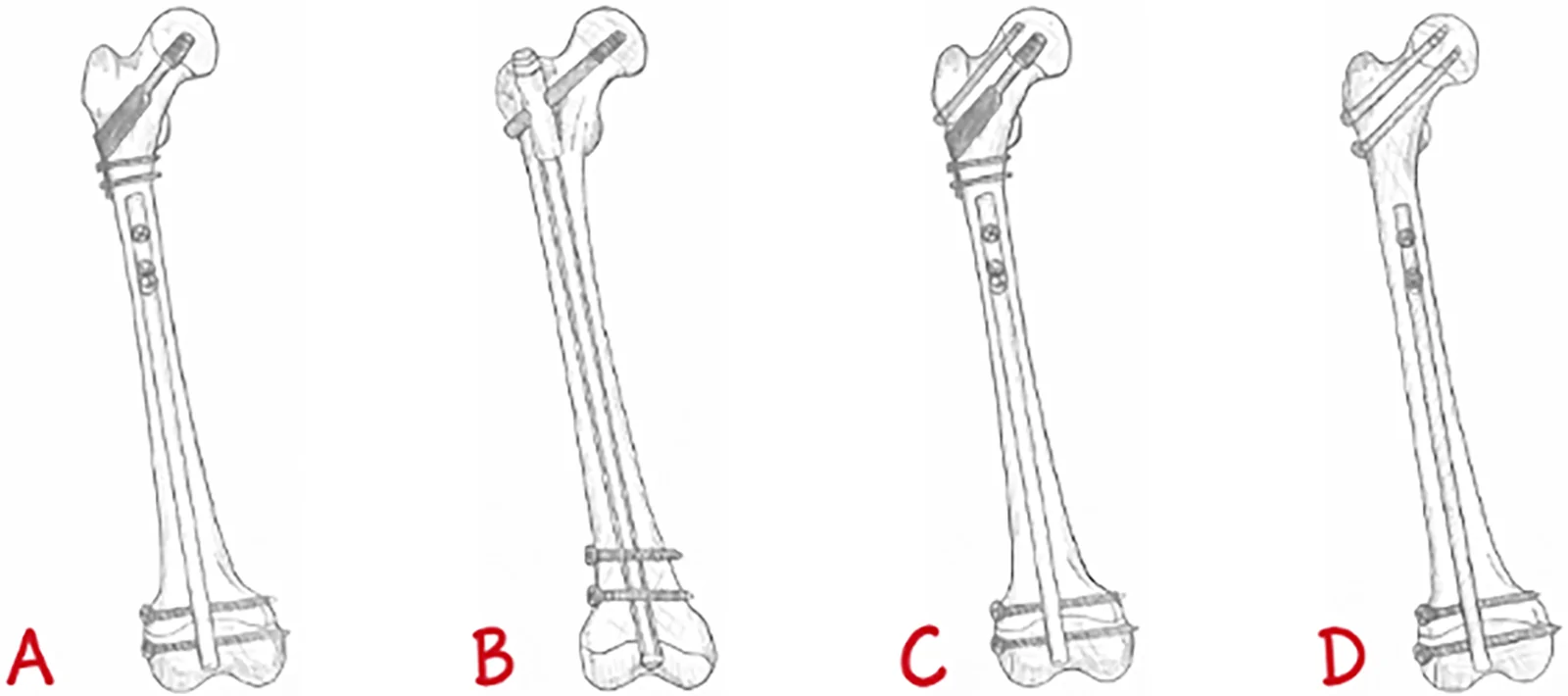
Implants used for fracture treatment, ordered by frequency. A: dynamic hip screw combined with retrograde femoral nail (*n* = 27); B: long cephalomedullary nail (*n* = 13); C: dynamic hip screw with anti-rotation screw combined with retrograde femoral nail (*n* = 7); D: cannulated screws combined with retrograde femoral nail (*n* = 5).

Proximal fracture outcomes were analyzed separately according to fracture pattern. Among the 28 patients with femoral neck fractures, six patients died and were not considered evaluable for late radiographic outcomes. All 22 evaluable femoral neck fractures achieved union. No cases of proximal nonunion, femoral head avascular necrosis, varus malunion, loss of reduction, implant failure, cut-out, or reoperation attributable to a mechanical complication of the proximal fracture were identified during the available follow-up.

Among the 24 patients with intertrochanteric fractures, one patient died, and 23 were evaluable for late radiographic outcomes. All 23 fractures eventually achieved union. One patient developed delayed union of the proximal fracture, which subsequently consolidated without reoperation. No intertrochanteric nonunion, varus malunion, loss of reduction, cut-out, or proximal implant failure was observed. Importantly, all three nonunions identified in the overall cohort involved the femoral shaft rather than the proximal fracture.

Overall, 17 of 52 patients (32.7%) experienced at least one major complication. All-cause mortality was the most frequent major complication (7 patients; 13.5%), followed by deep SSI (6 patients; 11.5%). Three patients (5.8%) developed nonunion and two (3.8%) developed delayed union. All three nonunions involved the femoral shaft. Of the two delayed unions, one involved the femoral shaft and the other involved the proximal intertrochanteric fracture; both subsequently achieved union. Symptomatic PE occurred in two patients (3.8%), both with fatal outcomes. Secondary outcomes included superficial SSI in five patients (9.6%) and DVT in one (1.9%). Unplanned reoperation was required in eight patients (15.4%). Six patients underwent procedures for infection management and three underwent revision procedures related to nonunion or failure of healing; these categories were not mutually exclusive because one patient presented both deep SSI and femoral shaft nonunion.

Among the seven deaths, five (71.4%) were attributed to systemic causes unrelated to local femoral fracture complications: pulmonary sepsis in three patients (42.9%) and systemic inflammatory response syndrome related to polytrauma in two (28.6%). The remaining two deaths (28.6%) were caused by pulmonary thromboembolism. None of the deceased patients developed deep SSI, and no death was considered directly attributable to the femoral fractures or the orthopedic procedures. Detailed complication distribution is presented in Table 1.

**Table 1:** Incidence and distribution of complications.

Category	Outcome	*n* (%)
Primary outcome	Any major complication	17 (32.7)
	All-cause mortality	7 (13.5)
	Deep surgical site infection (deep SSI)	6 (11.5)
	Delayed union	2 (3.8)
	Nonunion	3 (5.8)
	Symptomatic pulmonary embolism (PE)	2 (3.8)**†**
Secondary outcomes	Superficial surgical site infection (superficial SSI)	5 (9.6)
	Deep vein thrombosis (DVT)	1 (1.9)
	Unplanned reoperation (total)	8 (15.4)
	– debridement for infection	6 (11.5)
	– revision for nonunion/failure of healing	3 (5.8)
Mortality causes	Pulmonary sepsis	3 (42.9)
	Systemic inflammatory response syndrome (SIRS)	2 (28.6)
	Pulmonary thromboembolism (PTE)	2 (28.6)

*Note.* Complications were not mutually exclusive; a single patient could present more than one event during follow-up. No deceased patient developed deep surgical site infection, and no death was directly attributed to the femoral fractures or the orthopedic procedures.

**†** Cases of PE coincided with death (pulmonary embolism as the cause of death).

Bivariate analysis identified three factors significantly associated with complications: smoking, longer time to definitive treatment, and higher ISS. The complication rate was significantly higher in smokers (78.6%) versus non-smokers (15.8%; *p* < 0.001). Mean time to treatment was greater in the complications group (11.5 ± 5.0 days) versus the no-complications group (7.3 ± 5.7 days; *p* = 0.008). ISS was significantly higher in the complications group (28.9 ± 14.4) than in the no-complications group (20.3 ± 8.9; *p* = 0.029). Major complications occurred in 10 of 23 patients with open fractures (43.5%) and in 7 of 29 patients with closed fractures (24.1%; *p* = 0.234, Fisher’s exact test). Age was not significantly associated with complications (*p* = 0.116). Complete bivariable analysis results are presented in Table 2.

**Table 2:** Bivariable analysis of factors associated with major complications (primary composite outcome).

Variable	With complication (*n* = 17)	Without complication (*n* = 35)	*p*
Age (years), mean ± SD	46.8 ± 14.8	39.3 ± 16.0	0.116
Injury Severity Score (ISS), mean ± SD	28.9 ± 14.4	20.3 ± 8.9	**0.029**
Time to definitive treatment (days), mean ± SD	11.5 ± 5.0	7.3 ± 5.7	**0.008**
Smoking, *n* (%)	11 (64.7)	3 (8.6)	**<0.001**
Open fracture, *n* (%)	10 (58.8)	13 (37.1)	0.234
Comorbidities, *n* (%)	5 (29.4)	6 (17.1)	0.470
Associated fracture, *n* (%)	6 (35.3)	13 (37.1)	1.000

*Note. Outcome*: presence of at least one major complication (deep surgical site infection, delayed union, nonunion, pulmonary embolism, or death).

*Statistical tests*: Mann–Whitney *U* test for continuous variables and Fisher’s exact test for categorical variables.

Deep SSI occurred in 4 of 23 patients with open fractures (17.4%) and in 2 of 29 patients with closed fractures (6.9%). This corresponded to a conditional OR of 2.79 (exact 95% CI 0.36–33.75; *p* = 0.387). The association between open fracture status and deep SSI is presented in Table 3.

**Table 3:** Association between open fracture status and deep surgical site infection.

Fracture status	Deep SSI, *n* (%)	No deep SSI, *n* (%)	Total
Open fracture	4 (17.4)	19 (82.6)	23
Closed fracture	2 (6.9)	27 (93.1)	29
Total	6 (11.5)	46 (88.5)	52

*Note.* Percentages were calculated within fracture-status groups. The association was assessed using a two-sided Fisher’s exact test. Conditional OR = 2.79; exact 95% CI 0.36–33.75; *p* = 0.387. SSI, surgical site infection; OR, odds ratio; CI, confidence interval.

Multivariable logistic regression identified independent predictors of major complications. The model was statistically significant (*p* < 0.001), demonstrated adequate calibration (Hosmer–Lemeshow test, *p* = 0.543), and had moderate explanatory capacity (Nagelkerke pseudo *R*^2^ = 0.560). Smoking (adjusted OR 19.24; 95% CI 2.78–133.04; *p* = 0.003) and higher ISS (adjusted OR per point 1.10; 95% CI 1.01–1.19; *p* = 0.027) remained independently associated with the primary outcome. Time to definitive treatment (*p* = 0.373) and age (*p* = 0.269) were not independently associated with major complications. Open fracture status was not entered into the multivariable model because it did not meet the prespecified bivariable screening criterion of *p* < 0.20 (*p* = 0.234). Detailed results are presented in Table 4.

**Table 4:** Multivariable logistic regression analysis for major complications (primary composite outcome).

Variable	Adjusted OR	95% CI	*p*
Smoking (yes)	19.24	2.78–133.04	0.003
Injury Severity Score (ISS), per point	1.10	1.01–1.19	0.027
Time to definitive treatment, per day	1.08	0.92–1.26	0.373
Age, per year	1.04	0.97–1.10	0.269

## Discussion

This study identified factors associated with complications in 52 patients with ipsilateral proximal and femoral shaft fractures, a rare and complex injury pattern typically related to high-energy trauma. Results showed that 32.7% experienced at least one major complication (deep SSI, delayed union, nonunion, PE, or death), with smoking and trauma severity (ISS) emerging as independent predictors of adverse outcomes. These findings underscore that patient evolution is strongly influenced by systemic, modifiable factors beyond orthopedic management.

The overall complication rate (32.7%) aligns with reports in the literature ranging from 25% to over 50%, depending on sample characteristics and outcome definitions [1, 7]. A systematic review reported delayed union and nonunion rates of 10–20% and infection rates typically below 10% [1, 7, 9]. Our cohort showed delayed union or nonunion in five patients (9.6%). All three nonunions involved the femoral shaft, whereas the two delayed unions – one diaphyseal and one proximal – subsequently achieved consolidation. However, combined superficial and deep infection rates were elevated at 21.2%, likely reflecting high trauma severity (mean ISS 23.1), open fracture prevalence (44.2%), and systematic documentation. Deep SSI alone occurred in 11.5%, exceeding the rates reported in some femoral fixation series [14] and highlighting the impact of high-energy trauma and compromised soft tissues.

The most significant finding was the independent association between smoking and complications. Smokers showed substantially increased adverse event risk, consistent with robust evidence that smoking impairs bone and soft tissue healing and increases infection and nonunion risk [15–18]. Nicotine causes vasoconstriction, reducing local perfusion, while carbon monoxide decreases oxygen delivery, creating hypoxia that impairs osteoblastic function and callus formation [15, 16]. Systematic reviews demonstrate consistently increased complication risk in smokers [15, 17], with recent studies suggesting nicotine exposure alone may increase postoperative complications [18]. However, the wide confidence interval for smoking’s OR suggests imprecision related to sample size; the association direction is consistent and clinically relevant, but the exact magnitude requires cautious interpretation.

The ISS was an independent predictor of complications, with progressive risk increase as trauma severity increased. This is expected, as ISS reflects polytrauma physiological burden and correlates with organ dysfunction, systemic inflammation, infection susceptibility, and thromboembolic/respiratory complications.

The mortality pattern was predominantly determined by systemic and trauma-related complications. Five deaths were attributed to pulmonary sepsis or systemic inflammatory response syndrome related to polytrauma, whereas two were caused by pulmonary thromboembolism. None of the deceased patients developed deep SSI, and no death was directly attributed to the femoral fractures or the orthopedic procedures. Nevertheless, the fatal thromboembolic events reinforce the importance of thromboprophylaxis in patients with severe trauma, lower-extremity fractures, and prolonged immobilization.

The mean interval of 8.7 days to definitive fixation was longer than the early fixation intervals generally recommended for femoral fractures [19–21]. Most patients in our cohort (82.7%) underwent staged management with temporary external fixation, whereas 17.3% received definitive fixation at the time of initial surgery. The decision to use a staged approach was individualized and considered the severity and high-energy nature of the trauma, the need to prioritize other injuries and surgical procedures, soft-tissue conditions, and operating-room prioritization in a high-volume tertiary trauma referral center. These findings provide clinical and institutional context for the observed interval but should not be interpreted as evidence that delayed fixation is harmless. Although time to definitive treatment was associated with complications in the bivariable analysis, it was not independently associated with the primary outcome after adjustment. Given the limited sample size, residual confounding, and the absence of standardized patient-level documentation of the specific reason for postponement, a causal relationship between treatment timing and complications cannot be established.

Open fractures were associated with numerically higher rates of both the primary composite outcome (43.5% versus 24.1%) and deep SSI (17.4% versus 6.9%). However, these associations did not reach statistical significance (*p* = 0.234 and *p* = 0.387, respectively). For deep SSI, the point estimate suggested higher odds among patients with open fractures, but the exact confidence interval was wide (OR 2.79; 95% CI 0.36–33.75), reflecting the small number of infection events and limited statistical precision. Therefore, the absence of statistical significance should not be interpreted as evidence that open fracture status has no effect on infection risk. The present findings remain inconclusive and may also be influenced by residual confounding inherent to the retrospective design.

The proximal fracture represents a particularly important component of this combined injury pattern because missed diagnosis, inadequate reduction, and fixation failure may result in nonunion, varus malunion, or femoral head avascular necrosis. In the present cohort, all 45 proximal fractures available for late radiographic assessment eventually achieved union. All 22 evaluable femoral neck fractures united without documented avascular necrosis, varus malunion, loss of reduction, or implant failure. One intertrochanteric fracture showed delayed union but subsequently consolidated without reoperation. Furthermore, all three nonunions observed in the cohort involved the femoral shaft rather than the proximal fracture.

Although these proximal outcomes were favorable, they should be interpreted cautiously. Seven deaths reduced the number of patients available for late radiographic assessment, and the retrospective design did not allow standardized assessment of reduction quality or serial radiographic evaluation at uniform intervals. In addition, avascular necrosis may become apparent after prolonged follow-up. Therefore, the absence of this complication during the available follow-up does not exclude the possibility of later presentation. Early recognition of the proximal fracture, anatomical reduction – particularly for femoral neck fractures – and a fixation strategy that avoids conflict between the proximal and shaft implants remain essential principles of treatment [1, 6, 8, 9].

Although the literature frequently discusses single versus dual implants for bifocal femur fractures [8, 9], this study lacked power to compare fixation strategies as complication determinants given limited events and heterogeneous fracture patterns. Systemic factors (trauma severity and smoking) appear central to overall complication burden and should guide therapeutic planning and risk stratification.

This study has inherent retrospective limitations, including dependence on medical record quality, potential event underreporting, and heterogeneous complication documentation. Although the sample size limits the detection of smaller associations and leads to wide confidence intervals for some estimates, the present cohort is, to the best of our knowledge, the largest single-center series published on ipsilateral proximal and femoral shaft fractures. Additionally, the specific patient-level reasons for postponing definitive fixation, including physiological parameters, intensive care unit admission, and preceding surgical procedures, were not consistently recorded in a standardized manner and could not be quantified retrospectively. Cause-of-death classification was based on the information documented in the medical records and does not exclude an indirect contribution from the overall trauma burden, immobilization, or treatment.

Notably, the two largest cohorts available in the literature were multicenter studies, including 140 patients from three level I trauma centers reported by Yeager et al. and 89 patients from eight trauma centers reported by Cannada et al. [22, 23]. Therefore, in addition to providing data on a rare fracture pattern, this study presents a single-center experience with relatively consistent treatment pathways and follow-up practices. Nevertheless, it describes the complication burden in this setting and found an independent association of smoking and ISS with adverse outcomes after multivariate adjustment.

In conclusion, complications are not infrequent in this rare and complex fracture pattern. Smoking and elevated ISS were independent predictors of complications after surgical treatment, reinforcing the importance of risk stratification and targeted perioperative measures.

## Conclusion

In surgically treated ipsilateral proximal and femoral shaft fractures, smoking and a higher ISS were independently associated with the occurrence of complications. In this fracture pattern, major complications affected approximately one third of the cohort.

All proximal fractures available for late radiographic assessment achieved union, with no documented femoral head avascular necrosis, proximal nonunion, varus malunion, or proximal implant failure during the available follow-up. Nevertheless, the sample size, the number of deaths, and the retrospective follow-up limit definitive conclusions regarding uncommon or late proximal fracture complications.

## Data Availability

The data supporting the findings of this study are available from the corresponding author upon reasonable request.
